# Predicting the in-game status in soccer with machine learning using spatiotemporal player tracking data

**DOI:** 10.1038/s41598-022-19948-1

**Published:** 2022-09-29

**Authors:** Steffen Lang, Raphael Wild, Alexander Isenko, Daniel Link

**Affiliations:** 1 Department of Sport and Health Sciences, Technical University of Munich, Munich, Germany; 2 Department of Informatics, Technical University of Munich, Munich, Germany; 3 Munich Data Science Institute (MDSI), Technical University of Munich, Garching, Germany

**Keywords:** Engineering, Mathematics and computing, Computational science, Computer science, Software

## Abstract

An important structuring feature of a soccer match is the in-game status, whether a match is interrupted or in play. This is necessary to calculate performance indicators relative to the effective playing time or to find standard situations, ball actions, and other tactical structures in spatiotemporal data. Our study explores the extent to which the in-game status can be determined using time-continuous player positions. Therefore, to determine the in-game status we tested four established machine learning methods: logistic regression, decision trees, random forests, and AdaBoost. The models were trained and evaluated using spatiotemporal data and manually annotated in-game status of 102 matches in the German Bundesliga. Results show up to 92% accuracy in predicting the in-game status in previously unknown matches on frame level. The best performing method, AdaBoost, shows 81% precision for detecting stoppages (longer than 2 s). The absolute time shift error at the start was ≤ 2 s for 77% and 81% at the end for all correctly predicted stoppages. The mean error of the in-game total distance covered per player per match using the AdaBoost in-game status prediction was − 102 ± 273 m, which is 1.3% of the mean value of this performance indicator (7939 m). Conclusively, the prediction quality of our model is high enough to provide merit for performance diagnostics when teams have access to player positions (e.g., from GPS/LPM systems) but no human-annotated in-game status and/or ball position data, such as in amateur or youth soccer.

## Introduction

Match events are a fundamental component of soccer performance analysis^1^. Competition information providers (CIP) collect in professional soccer more than one thousand events per match, e.g., standard situations, passes, shots, dribbles, fouls, and the ball status (= ball goes out of play or comes into play). Based on this data, coaches develop match strategies, media companies enhance their coverage, and science explores the performance structure in soccer. The event data collection is usually done by human data loggers and therefore takes much time and effort. Hence, this data exist only for professional matches, less so during training or for amateurs.

Recent years have seen the rise of position-detection systems, which introduced a second type of information: spatiotemporal tracking data. Although semi-automatic optical tracking systems have been used to track player positions in professional matches^2^, cheaper and more obtainable automatic tracking systems, using global positioning systems (GPS) or radar-based local positioning measurement systems (LPM)^3^, are commonly used for training and amateur matches. However, collecting ball position data is only possible using optical tracking systems, but these need high manual operation effort and are therefore only available in professional soccer. Recently, some LPM systems provide balls with integrated sensors (e.g., Kinexon Corp.), but it is not common in soccer so far. Therefore, the vast majority of teams lack ball position information and the ball status since they use GPS tracking^4^.

However, the ball status is one of the most basic information during a soccer match and a fundamental prerequisite for match analysis. There are two reasons: First, the amount of time where the ball is in-play within a match varies greatly, and so are the intensity and distances covered by players^5,6^. Hence, performance indicators for longitudinal analysis must be calculated relative to the effective playing time. Second, the ball status is essential in order to interpret player activity relative to the match context. Science has proposed numerous approaches for extracting advanced tactical concepts from positional data, for instance, offensive tactical performance^7^, attacking style^8^, synchrony between teams and players^9^, expected position value^10^, or pressing behavior^11^. Without the ball status, it is impossible to derive meaningful performance indicators from these concepts.

In this paper, we address this lack of ball status data and metrics based on it and build a machine learning (ML) model to derive this information. Since ball positions are not easily available, we used only player positions, which are affordable to collect and have high accuracy^12^. We hypothesize that the ball status and other events can be derived from these data because player movement behavior, e.g., speed, acceleration, orientation, depends on ball status. Our approach contributes to three real-world problems: First and most important, the undeveloped performance analysis in professional soccer teams’ training or semi-professional soccer matches. Here, player position collection with GPS or LPM systems is common, but no manual ball status or event logging. Providing the ball status solely from a low-cost tracking system, e.g., GPS, empowers all teams from youth and amateur leagues in their analyses without high investments. Second, our approach can support CIPs ball status logging, because it is only one of many data logger tasks, and particularly in live situations cognitively challenging. Providing a ball status estimation, the operator must not detect each ball status toggle, but only correct wrong algorithm assignments. This hastens the process and reduces mental stress. Third, the ball status is a precondition for detecting events based on positional data. Without, it is impossible to differentiate between a pass during a match and kicking the ball to the set piece execution spot within an interruption. Our approach provides a component without any human interaction, which can be used for the event detection processing pipelines based on, e.g., broadcasting video feeds.

Until now, no studies have reported the estimation of the in-game status solely using player data in high-frequency output. There are studies on deriving events from soccer data, i.e., video streams, event data, and tracking data. Although most previous research focused on the automatic detection of events in video streams using computer vision methods^13–15^, we focused on spatiotemporal tracking data. Gudmundsson and Wolle^16^ extracted ‘ball-in’ and ‘ball-out’ events using both player and ball data. Wei et al.^17^ performed the same research using an ML approach as an interim step for further analyses. They fed a decision tree classifier with players or ball positions to detect the ball status in 4 s chunks. Subsequently, they used this generated information to analyze highlights and non-highlights and set-piece types for sequences of in-play and stoppages, respectively. Richly et al.^18^ used player and ball tracking data of the German Bundesliga to detect passing events consisting of a kick and a reception. They trained a neural network of three layers with a single output neuron and showed that it detects events well. Despite extracting events with ML methods, Link and Hoernig^19^ proposed a heuristic model using ball and player positions. They extracted the individual ball possession (IBP) of players to evaluate the time a player has the ball under his control. They stated that IBP is an important prerequisite for further analyses and performance indicators^20^, since, without it, determining events that happen and assigning them to players is impossible. In a follow-up study, Link and Lang^21^ applied IBP to extract ball actions (events of a player with the ball) from spatiotemporal data. They used IBP, field position, player trajectories, and ball status to classify the IBP, e.g., standard situation, cross, or pass. More recently, but similarly, Vidal-Codina et al.^22^ extracted match events from positional data. Summarizing, each of these studies used ball positions and status, and consequently are not yet applicable to amateur teams.

Against this background, our study evaluated whether the ball status can be determined from player positions only. Therefore, we apply four different well-known ML algorithms: decision trees, random forests, AdaBoost and logistic regression. The ability to find higher order relations within data and to extract information brings ML into a good position to solve the lack of ball status data. Our dataset comprises positional information of all players on the field in 102 matches of the German Bundesliga, including the ball status, which serves as the ground truth to train and test the ML models. This study is designed two-fold: First, we tested the ability of these four well-established ML models to predict the ball status by evaluating them using (I) frame-by-frame prediction and (II) stoppage prediction. Second, we investigated whether these models can be used in soccer performance analyses. Hence, (III) for video analysis tasks, we considered the time shift error to the real stoppages starting and ending points depending on their type (e.g., Free kick, Throw-in) and (IV) we evaluated the application of the estimated ball status for deriving performance indicators.

## Methods

### Dataset

This study used 102 German professional soccer Bundesliga (82) and Bundesliga 2 (20) matches from 31 of 34 rounds of the season 2017/18. Each match data contains the positional data of players (x/y) and ball (x/y/z) recorded in 25 fps, event data, and basic match information, e.g., player/team names, and playing positions. Positional data was collected semi-automatically using an optical tracking system (TRACAB, Chyronhego, NY) and in-game information was collected manually by human observers^23^. The accuracy of the tracking system was validated by Linke et al.^12^. All 36 teams are represented with a minimum and maximum of two to twenty matches. Each team had home and away matches, except one which had only away matches available. We split the dataset into training (45 matches), validation (10), and test (47) sets with only Bundesliga matches considered in the training and validation set. Consequently, all Bundesliga 2 matches were included in the test set. To analyze our dataset in terms of class distribution, we compared the number of stoppages per match, total duration, and stoppage types (Kick-off, Goal kick, Free kick, Corner kick, Penalty kick and Throw-in) with other studies^24,25^ and found them to be similar. The dataset split was designed in a stratified fashion so that the number of stoppages, their length, and their types occurred with similar probability in each split.

### Models

Four ML algorithms were used: logistic regression (LR), as a baseline binary classifier, and three tree-based classifiers: decision trees (DT), random forests (RF), and AdaBoost (AD), as they have only a few hyperparameters to train and are known for good results^26,27^. For the experiments here, the implementations for these algorithms were provided using the scikit-learn library (v0.20.3) for Python^28^. We ran our experiments on a 32-core processor with 64 GB RAM. Apart from the LR training, no limit on training time or the number of iterations to stop a run was assigned. Thus, the algorithms ran until they terminated naturally. For LR, a maximum number of interations was set, which was left as the default (100).

### Data preprocessing

From the raw data, we derived a set of features for each time frame in a match with a frequency of 25 fps. First, we reduced the dataset for each frame to only contain data for the 22 players on the pitch. When less than 22 active players are present, the remaining values are filled with zeros. The players were sorted according to the match sheet, which depends on the players’ position and whether they play for the home team. The spatial coordinates of all players were flipped in the second half to reverse side switching after halftime. Additionally, training data were augmented by switching the home and away team information because home team players are always listed first. This doubles the available training data and removes the home advantage, which could influence the predictions. Each sample now contains information for 22 players and their positions, speed, and acceleration.

Next, we augment each sample with features from the past and future frames to provide more context information to the models. More precisely, each sample contained additional information of 0.4, 0.8, 2, 4, 20, 40, and 80 s frame from both the past and the future. The reason for this choice of values is to provide information about the players’ actions immediately before and after the current situation as well as some long-term context, leaving the selection of important information to the learning algorithm. We evaluated their impact on the section ‘Evaluation of ML models’.

We provided unscaled data for the DT, RF, and AD models. The values of each feature in LR were linearly transformed such that their empirical mean and standard deviations are zero and one, respectively. Decision trees, however, are based on a comparison of values^29^. Since the aforementioned transformation does not change the order of values, it does not affect the predictions of a decision tree. Hence, normalization is not applied to DT, RF, and AD.

Summarizing, all models used the same input features: the basic feature set for each frame sample was enhanced by 14 time shifts, both 7 from the past and future. And the four individual features of all 22 players on the field are each player’s *x*- and *y*-coordinate in meters, velocity in m/s, and acceleration in m/s^2^. Resulting in each sample consisting of 1320 (15 × 4 × 22) input features.

### Hyperparameter search

Each learning algorithm requires parameterization that influences the training process and significantly affects performance. Since there is no one-fits-all configuration for a single model, we ran a hyperparameters search for each model individually. For our study, we used a grid search for LR and AD and a random search^30^ for RF and DT due to their several hyperparameters configurable via the scikit-learn library^28^. All configured parameters of the hyperparameter search, as well as the best candidates, are described in detail in Appendix.

Another hyperparameter, as a part of postprocessing, is the kernel size of the median filter (see ‘Evaluation of ML models’),which is model-independent and is applied for stoppage evaluation only. That is, the search for the optimal kernel size is performed for each model with the best-found hyperparameters for frame-by-frame prediction. The inspected range of kernel sizes is from 1–901 frames.

### Evaluation

We evaluated the performance of the final models using four approaches. First, a *frame-wise* evaluation, which compares the ball status in the ground truth to the prediction in each frame. As performance metrics, we computed the accuracy and F1-Score for each model. Furthermore, we compared the prediction to a random guessing approach per match by taking the percentage of in-game frames, we refer to this as the knowledge gain. It is calculated for each match using the prediction accuracy minus the percentage of in-game frames. However, this frame-wise performance does not necessarily translate into correctly identified match interruptions since all consecutive frames must have the same value for a stoppage. For example, a single ball-in detection in a long streak of ball-out labels creates two stoppages instead of a single one.

Consequently, and secondly, model performance was evaluated *stoppage-wise*. The basic idea is to extract stoppages from the original data and predictions by searching for a matching pair between them. Stoppages are extracted by identifying the frames at which the match was interrupted and resumed, where the first and last frames with the ball-out label define the start and end of a stoppage, respectively. A suitable metric to compare predicated stoppages with actual ones is the intersection over union (IoU), which is common in object detection benchmarks^31,32^. The IoU is computed for a pair of real and predicted stoppages as the overlap time between them divided by the overall time covered by both stoppages, i.e., the overlap time plus the sum of the non-overlapping durations. For our paper, two stoppages are matched if their IoU is at least 50%, which guarantees that each real stoppage is matched only with one predicted stoppage and vice versa.

A good model should apply to analysis tasks. Hence, the results of performance metrics using the ball status prediction must be comparable to those using real data. The IoU metric assigns a predicted stoppage to the corresponding ground truth interruption, even when the overlap is imperfect. Subsequently, a shift in the correct starting and ending points for each stoppage exists, which affects its application in video analysis tasks.

Third, we checked whether the predicted stoppages’ starting and ending points did not differ much from the ground truth. We calculated the shifts between real and predicted points for the start and end. A fundamental task in video analysis is to analyze standard situations, thus, we assumed that our predictions should be in a range within ± 2 s. In that case, a practitioner could easily find and add time marks to analyze the execution of a standard situation.

Fourth, we evaluated the quality of the performance indicator Total Distance Covered (TDC) in the effective playing time (TDC_E_). TDC is one of the most common performance indicators for estimating workload^33–35^. TDC_E_ represents the running activity when the ball is in-play and can be interpreted as the match intensity. Since the error in predicting the ball status introduces an error to TDC_E_, we checked whether this error is acceptable for performance analysis. Therefore, we calculated TDC_E_ for each player three times per match using the ball status based on (1) ground truth, (2) AD prediction and 3) a naïve approach and compared them. For the naïve approach, we calculated an approximation for TDC_E_ by taking the real TDC by the player for the whole match and the mean percentage of the effective playing time in all matches. In our test matches, the mean percentage of the effective playing time was 59.6 ± 6.0% (Min. = 47.4%, Max. = 69.7%). To reduce noise, only field players who played a full match were included in the analysis.

## Results

### Evaluation of ML models

#### Frame-by-frame prediction accuracy

Table 1a shows the average F1-Score and accuracy of our models against all 47 test matches. Here AD shows the best results in accuracy (ACC) with 0.92 ± 2.1 and F1-Score with 0.93 ± 2.4 overall. The other models ranged from ACC = 0.87–0.91. The individual match prediction accuracies for each model are described in detail in Appendix. AD showed the best accuracy in 44 of 46 matches. Its prediction outperforms the naïve approach and the knowledge gain ranges from 0.20 to 0.44 (Mean = 0.32 ± 0.05).

**Table 1:** Prediction results for A) frame-wise and B) stoppage-wise prediction of the four chosen ML models in 47 test matches.

	Logistic regression	Decision tree	Random forest	AdaBoost
**A) frame-wise prediction**				
Accuracy	86.7 (± 2.0)	84.6 (± 1.9)	89.3 (± 2.0)	**92.0 (± 2.1)**
Precision	88.3 (± 3.9)	85.8 (± 4.3)	88.1 (± 4.0)	**93.4 (± 2.6)**
Recall	89.4 (± 3.4)	88.8 (± 3.0)	**94.6 (± 2.2)**	93.0 (± 4.0)
F1 Score	88.7 (± 2.3)	87.2 (± 2.3)	91.2 (± 2.3)	**93.1 (± 2.4)**
**B) stoppage-wise prediction**				
Precision	58.9 (± 7.6)	68.6 (± 7.2)	68.8 (± 6.4)	**81.1 (± 7.4)**
Recall	64.5 (± 6.7)	61.6 (± 6.4)	67.4 (± 5.6)	**78.9 (± 5.7)**
F1-Score	61.2 (± 6.1)	64.7 (± 5.8)	67.9 (± 5.1)	**79.8 (± 5.8)**

Accuracy, Precision, Recall, and F1-Score in percent with standard deviation. In B) only stoppages with a minimum duration of 2 s are considered. The best results are bold.

To better understand what information in the input data influences a model’s decisions, there are methods to identify the importance of each feature^30^. We used the random forest feature importance, where the importance of a feature is computed as the information gain in each split based on this feature and weighted by the amount of training data reaching this split. We focused on the estimates of feature importance in the final AD model since it had the best accuracy. The above metric applies to all RF methods, including AD^36^.

**Figure 1:**
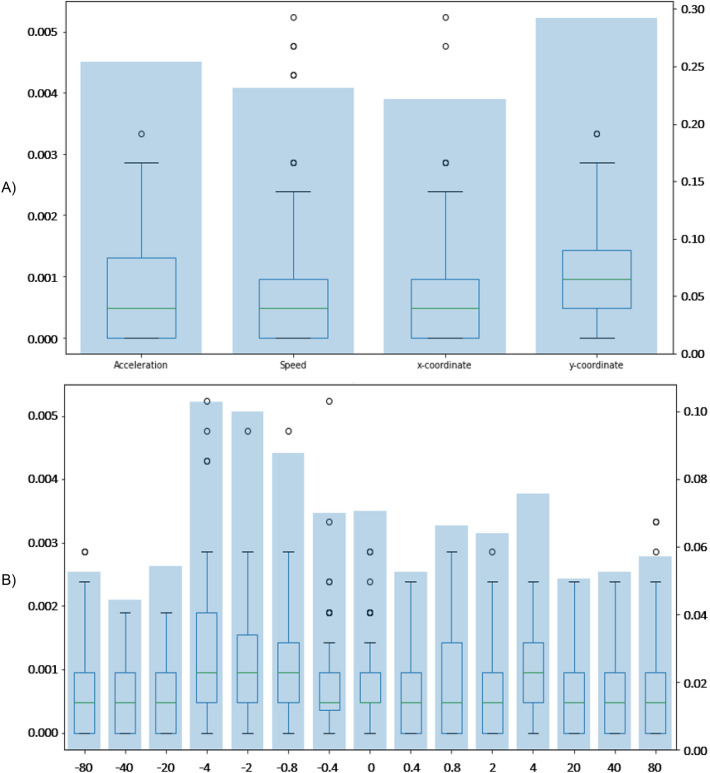
Boxplots (left y-axis) and sums (right y-axis) of mean decrease in impurity per feature in the feature class (**A**) Type of value and (**B**) Time shift in seconds.

For more insightful feature analysis results, we group in Fig. 1 the features by their feature class, e.g., type of value and time shift. Feature importance was defined for each value between zero and one, meaning that the higher the value, the higher the influence, which all together sums to one. Figure 1a) provides evidence that no single type of value trivially determines the model’s output, but the decision is spread out over all value types. Figure 1b) shows the features grouped using the associated time shift. Again, considerable importance was assigned to each group, meaning that all features are important for prediction. Nevertheless, there is a trend toward data being more important if it is close to the prediction point. However, the model puts no particularly strong weight on features referring to the exact time of the prediction (time shift = 0). This indicates that the model takes more information from the coarse situation than from the precise moment. Additionally, within 4 s around the prediction point, more importance was assigned to features referring to past moments.

#### Stoppage prediction accuracy

As a second evaluation step, we tested whether the models can predict stoppages as a sequence of frames where the ball is out of play. To create stoppages from our prediction model, we use a median filter. The filer is parametrized to a window of 3.64 s (i.e., 91 frames) to smoothen the raw ball status predictions and afterwards we computed IoU to the ground truth stoppages. This window has been empirically evaluated on the validation set to maximize the F1-Score. We excluded stoppages shorter than 2 s from our analysis since they are considered unimportant to performance analysis and concern only 1.3% of all stoppages. Additionally, we focused on the AD model as it shows by far the best results in F1-Score for the 2 s length for further analyses. AD has a 12% higher mean F1-Score overall matches (F1 = 0.80 ± 0.06) compared to the other three models ranging from 0.68 ± 0.05 (RF) to 0.61 ± 0.06 (LR) (Table 1b). AD surpassed their stoppage predictions in 42 of 47 matches, as described in the Appendix.

To check whether the selected AD model has a bias in its prediction when working with different leagues, we split the test set into the first and second league. The results show almost no difference between the leagues average F1-Scores (Bundesliga: F1 = 0.80 ± 0.05, Bundesliga 2: F1 = 0.79 ± 0.07). This speaks to the generalizability of our model.

### Evaluation of application for performance analysis

#### Starting and ending point prediction accuracy

**Figure 2:**
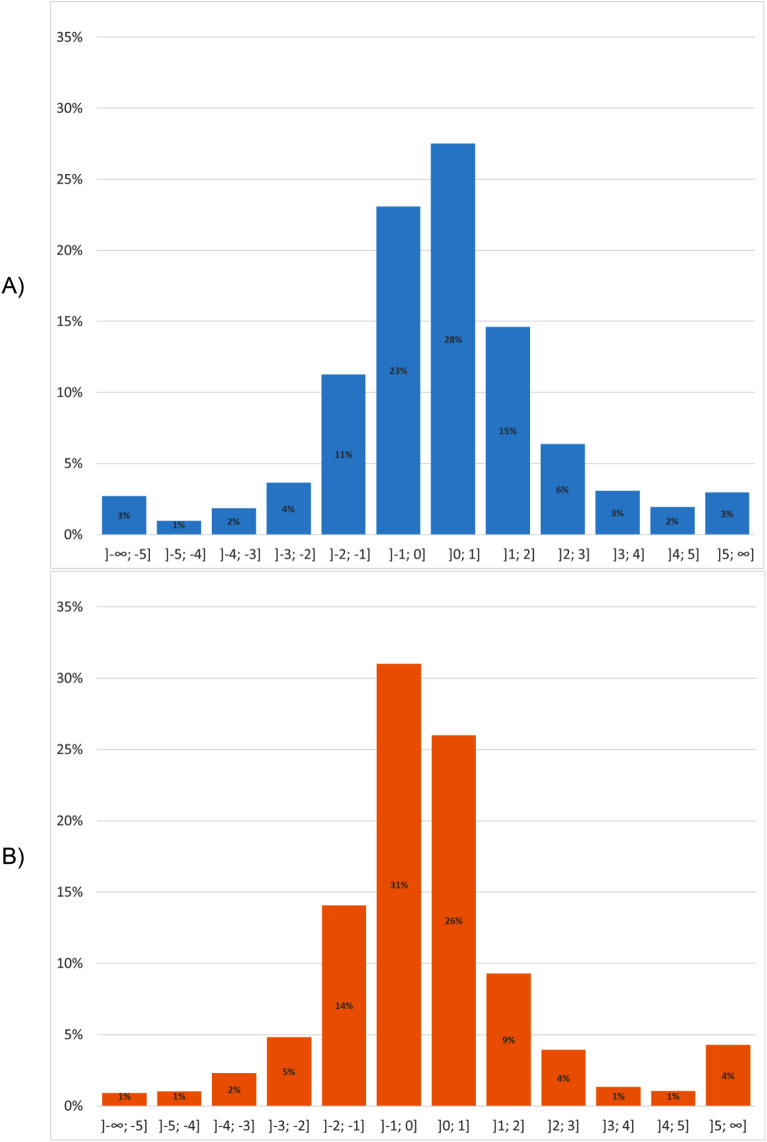
Deviation of time shifts for (**A**) starting and (**B**) ending point prediction with AdaBoost model for all stoppages found in 47 test matches. In 77% (start) and 81% (end) the shift is between ± 2 s.

It is important to have information on the position of stoppages in the video footage for video analysis. First, we split all correct stoppages (true positives) into four groups according to their total length because we assumed that prediction accuracy depends on stoppage length. We differentiated between the groups with less than 10, 20, 30, and equal or more than 30 s. However, no significant differences between these four groups were found. Second, we checked the length of shifts. Here, in more than 51% (start) and 58% (end), the deviation from the real point was $\le$ 1 s, and more than 78% (start) and 80% (end) of all stoppages had $\le$ 2 s shift (Fig. 2). The mean absolute shift was 1.79 ± 3.44 s (99% CI [1.65, 1.94]) for the starting points and 1.68 ± 3.47 s (99% CI [1.54, 1.83]) for the ending points.

Next, we checked whether the model predicted specific interruptions better. Here, we compared all six stoppage types (Kick-off, Goal kick, Free kick, Corner kick, Penalty kick, and Throw-in) using their recall for our best model with the minimum duration of 2 s. Note, a stoppage is type ’Kick-off ‘ when the match proceeds with a Kick-off after the stoppage. Additionally, we checked whether the model has problems detecting the starting and ending points of specific types (Table 2). The recall varies between the types from 0.92 (Kick-off) to 0.74 (Throw-in). The mean absolute start and end shifts differ only slightly within four types (Corner kick, Goal kick, Throw-in, and Free kick) between 1.42–2.00 s (start) and 1.54–1.87 s (end). However, that is not the case for the Kick-off and Penalty kick. This is attributed to their low frequency in our dataset and longer stoppage duration.

**Table 2:** Prediction quality per stoppage type for 3886 correct predicted stoppages in 47 test matches with AdaBoost and minimum stoppage length of 2 s.

Stoppages				Absolute shift [s]							
Type	*n*	Length	Prediction	Start				End			
		Mean [s]	Recall [%]	Mean (SD)	Median	99% CI	Min./Max.	Mean (SD)	Median	99% CI	Min./Max.
Overall	3886	21.4	78.9	1.79 (± 3.44)	1.00	[1.65, 1.94]	0.00/69.08	1.68 (± 3.47)	0.84	[1.54, 1.83]	0.00/53.88
Kick-off	153	50.2	91.6	4.80 (± 9.09)	2.60	[2.89, 6.72]	0.04/69.08	0.90 (± 1.21)	0.56	[0.65, 1.16]	0.00/8.68
Penalty	13	61.7	86.7	5.41 (± 13.36)	1.04	[− 5.91, 16.73]	0.2/49.48	10.50 (± 18.62)	0.72	[− 5.28, 26.27]	0.04/53.00
Free kick	1290	26.9	81.3	1.80 (± 2.99)	1.04	[1.58, 2.01]	0.00/37.00	1.87 (± 3.78)	1.00	[1.60, 2.14]	0.00/53.88
Corner kick	367	26.9	80.7	1.58 (± 3.04)	0.88	[1.17, 1.99]	0.00/34.72	1.81 (± 4.49)	0.68	[1.20, 2.41]	0.00/32.56
Goal kick	568	23.0	79.8	2.00 (± 4.09)	0.88	[1.55, 2.44]	0.00/49.12	1.55 (± 2.52)	0.92	[1.28, 1.83]	0.00/33.08
Throw-In	1495	13.0	74.3	1.42 (± 1.85)	0.92	[1.30, 1.55]	0.00/25.52	1.54 (± 2.82)	0.80	[1.36, 1.73]	0.00/37.40

Absolute time shift to the ground truth of predicted stoppages with AdaBoost.

#### Performance indicator accuracy

Table 3 compares TDC_E_ calculated using the ball status based on ground truth, AD prediction, and a naïve approach by reporting common error indicators (mean error, mean absolute error (MAE), 99CI, R (Pearson), ICC (two-way random model). Results show that the differences between the TDC_E_ of both ground truth and AD are smaller than the differences between the TDC_E_ of both the ground truth and naïve approach. For instance, the mean error of AD TDC_E_ was − 102 m, which is 15.6 times smaller than the mean error introduced using the naïve approach (1,590 m) and 1.3% of the mean value of TDC_E_ based on the ground truth (7939 m). Similarly, correlation coefficients are much higher when using AD compared with the naïve approach.

**Table 3:** Total distance covered per player in the effective playing time calculated with in-game status data of ground truth, AdaBoost prediction and a naïve approach for 751 players that played only the full match and without goalkeepers.

	Total Distance Covered per player in effective playing time		
	Ground Truth (GT)	AdaBoost	NaÏve approach
Mean [m]	7939 (± 1631)	8041 (± 1683)	6348 (± 1099)
Mean error to GT [m]		− 102 (± 273)	1590 (± 683)
Mean absolute error (MAE) to GT [m]		238 (± 169)	1594 (± 674)
99% confidence interval (MAE) [m]		[222 ; 254]	[1531 ; 1658]
Inter class coefficient (ICC)		0.985	0.532
R (Pearson)		0.987	0.949

Differences between ground truth to AdaBoost prediction and the naïve approach.

To interpret the error size considering performance analysis (see Discussion), we used the so-called ‘smallest worthwhile change’ (SWC) introduced by Hopkins^37^. SWC refers to the smallest change of a performance variable within a series of measurements, which has practical meaning in sports. For soccer, the literature suggests an SWC of 20% of the within-athlete or inter-athlete variation of a running performance indicator^38^. Following this recommendation, SWC for TDC_E_ is 326 m and is calculated using the standard deviation of ground truth TDC_E_ (1631 m).

## Discussion

### Discussion of results

The first aim of this study was to test the ability of four ML models to predict the ball status. Therefore, we used only the positional data of players and fed them into ML algorithms with the assumption that the target information lies in the collective running behavior of the players. Our results support this belief and showed that all tested ML algorithms provided significantly better results than guessing the ball status with a naïve approach in the frame-by-frame prediction (question I) (Table 1a). AD achieved the best result with an F1-Score of 0.93 and a mean knowledge gain of 0.32 ± 0.05, whereas the other three models ranged from F1 = 0.87 (DT) to F1 = 0.91 (RF). Several promising results were obtained for predicting complete stoppages (question II) (Table 1b). Despite the good results of all ML models in the frame-wise prediction, AD by far produced the best results for the stoppage prediction, achieving an F1-Score of 0.80 ± 5.8 compared to the others ranging from 0.68 ± 0.05 (RF) to 0.61 ± 0.06 (LR). A deeper qualitative analysis detected some systematic flaws: First, for false negatives, there is a tendency to miss very short stoppages (2–4 s) of some types (Free kick, Throw-in). In these situations, either the median filter smooths too much, or the players’ behavior shows no difference as the match resumes promptly. The same smoothing problem occurs when a very short sequence of ball in-game lies between two stoppages, so the interruptions are stitched together. Second, is the problem of false positives, which can be seen within long interruptions where the players show more activity for a short period, in turn, leading the model to predict that the ball is in-game. This results in one correct and one false positive classified stoppage because the ground truth stoppage is assigned by the IoU metric to one of both and cannot be assigned twice. Overall, very short sequences of ball in-game or out-of-game are harder to detect. In these moments, the behavior of the players shows less differences, as they anticipate that the match will resume or interrupt rapidly. Studies on stoppage types show that the recall differs within types. For Throw-in and Corner kick, less recall is attributed due to the result of the median filter because these situations are prone to being followed by fast toggling ball status (Table 2).

A comparison of our results with other studies is possible only to a limited extent. To the best of our knowledge, there is only the study by Wei et al.^17^ that performs a prediction of the ball status. However, since they sliced the match into 4 s chunks and predicted whether a chunk was in-game, their task differs to ours. Nevertheless, they achieved a mean accuracy of around 90% with player position data and almost 100% with ball position data. While their results are exceptional, they did not clarify how they handle stoppages with a duration of less than 4 s. Furthermore, there is no mention of whether the prediction accuracy is only for ‘chunk detection’ or complete stoppages. Compared with our study, where we achieved an accuracy of 80% for complete stoppages and 93% for frame-wise recognition with 25 Hz frequency, our model outperformed the chunk detection with higher accuracy and frequency. We assume that they did not predict complete stoppages, as it was not discussed in the following analyses. Gudmunsson and Wolle^16^ achieved an F1-Score of 0.94 for ‘ball-out’ event detection, where the ball leaves the field. However, this was achieved under the consideration of the ball and player position data. Khaustov and Mozgovoy^39^ recognized events like passes and shots on goal, but included the ball position in their input feature space. They obtained an F1-Score ranging from 0.93–0.99 and 0.86–0.88 for successful and unsuccessful passes, respectively. Richly et al.^18^ predicted passes, consisting of a kick and a reception, but did not differentiate between the ball status. Also, they included the ball position in their input feature space.

The analysis of stoppages’ starting and ending point time shifts (question III), shows promising results, where the shift was in more than 78% of all stoppages between ± 2 s (Fig. 2). It is in the nature of the IoU metric, the longer the stoppage, the more shift error is possible. Nevertheless, checking the deviation of shifts versus the length shows no trend in whether the model predicts short or long stoppages better. This means that starting and ending points are predicted with the same accuracy within different stoppage lengths, making the model feasible for performance analysis, such as video analyses, because practitioners want to find standard situations in a video stream. For example, the performance analysis of a Free kick is driven by the execution and the behavior of players just before and after the kick. Furthermore, analysts search for tactical patterns in the opponent’s play after standard situations. Our model provides an opportunity to solve these tasks, because most of the ending points are estimated with an error of less than 2 s.

The last part of the analysis evaluates whether the accuracy of the estimated ball status is high enough to derive ball status-related performance indicators (question IV). Here, we used SWC by Hopkins^37^, who argued that a measurement device is ‘useful’ for measuring the performance variable if the random error (noise) of the device is below the SWC. When using the AD predicted ball status, the standard deviation of the TDC_E_ error, which represents the noise, was 273 m. This is lower than the SWC of 326 m (Table 3). Thus, the ball status estimation accuracy is sufficiently high to measure ball status-related performance indicators. The data indicate that the naïve approach would lead to a higher error since the standard deviation of the TDC_E_ error (683 m) was higher than the SWC, which makes the naïve approach unacceptable for calculating TDC_E_. This results from the fact that the speed of the players is much higher when the ball is in play than otherwise.

### Discussion of methods and limitations

To provide a basic foundation, we started by classifying each frame into a binary state. We chose four well-known classification algorithms to keep the workload and computing time in check. However, other classification algorithms, e.g., k-nearest neighbor or Naive Bayes, could also be applied and provide interesting results. It seems worth to try more complex ML algorithms like (recurrent) neural networks^18,40,41^ or change the input features to more complex ones. For our feature selection, we only selected the existing information, without generating new ones, to reduce computing time and keep it simple. However, different approaches that preserve the correspondences between features and players, and help with the generalization are conceivable. In our case, neither a specific feature nor value type class (x/y-coordinate, speed, acceleration, time shifts) shows a significant increase in importance compared with others (Fig. 1). We have shown that the time shifts around the prediction point [− 4 s, + 4 s] have higher feature importance for the ball status compared with other time shifts. Notably, the behavior of the players, seconds before the ball goes out or comes in, is more relevant for the model than the moment itself or the long-term information. Here, one could think about including trajectory heatmaps or vectors as input features to give the algorithm a better understanding on time depending behavior.

Adding more information around the events where the ball status swaps, e.g., the centroids of the teams^17^ or the centroid of the defense and attacking lines, could improve the prediction quality. Furthermore, it can be useful to introduce the essence of a stoppage into the algorithm, e.g., the minimum, maximum, distribution, and mean length. The feature corresponding to one player changes each time there is a substitution or the player is sent off. Instead, considering the playing position of each player gives the model a better understanding of which players may behave the same way in certain situations. Hence, it would be necessary to assign a defined playing position to each player data array after the match, which increases the effort for the analysts in post-match production.

We checked whether the model performance differed between leagues, which was not the case. One can argue that showing validity for the first and second leagues does not necessarily mean that the model works for lower leagues. However, we assume, that there are not too much differences which cause serious problems, but many similarities in general movement patterns, e.g., slowing down when the ball goes out-of-game and vice versa. Furthermore, we have not shown that our model works on other datasets. Nevertheless, we assume no problems by using data of other system classes since the position accuracy (LPM) and speed (GPS) are in a similar range^42^.

### Discussion of application

By providing the in-game status, our solution opens up the field for deeper and more detailed analyses for all teams that only have player tracking data (e.g., from GPS or LPM) without a dedicated ball position and no human data logger. Our solution provides the precondition for extracting higher tactical constructs from the positional data of players. Albeit the extraction of position data from broadcasting video feeds becomes easier in the future, our model is suitable for working with such data. Moreover, even with a rough ball position derived from a video feed, our model can be easily retrained to incorporate this information, thereby leading to better prediction quality^17^. We assume that including the ball position would lead the models to nearly 100% accuracy, as most of the interruptions, i.e., Kick-off, Corner kick, Penalty kick, and Throw-In, are by default depending on the ball position. More generally, we showed the applicability and potential of using ML on sports data. We believe the future will bring a completely automated extraction of events using a few low-cost data sources, like GPS tracking and a one-camera system. This will provide affordable deeper analyses for amateur teams. We believe our study is the first to use only the player position data to extract the ball status in high frequency, which is fundamental information about the ball and the match in soccer.

## Conclusion

Our data show that the trained AD model provides an appropriate technological solution for determining in-game status from time-continuous player positions only. The AD model can predict more than 78% of stoppages’ starting and ending points with a time shift error of less than 2 s, which should be good enough for video analysis tasks. By taking TDC as an example, our analysis shows that ball status-related performance indicators can be calculated with an acceptable error. In scenarios where teams have only access to player positions (e.g., from GPS/LPM systems) but not to a human-annotated ball status and/or ball position data, i.e., in amateur or youth soccer, our approach can enhance match analysis.

## Supplementary Information

Supplementary Information.

## Data Availability

The datasets generated and/or analysed during the current study are not publicly available due to the authors are not the owner of all data, but they are available from the corresponding author on reasonable request.
