# Supplementary material for: Predicting the in-game status in soccer with machine learning using spatiotemporal player tracking data
Source: Sci Rep. 2022 Sep 29;12:16291. doi: 10.1038/s41598-022-19948-1 (PMC9522646; doi:10.1038/s41598-022-19948-1)
Supplement: Supplementary file 1 — Supplementary Information. [file 41598_2022_19948_MOESM1_ESM.docx]

## Appendix


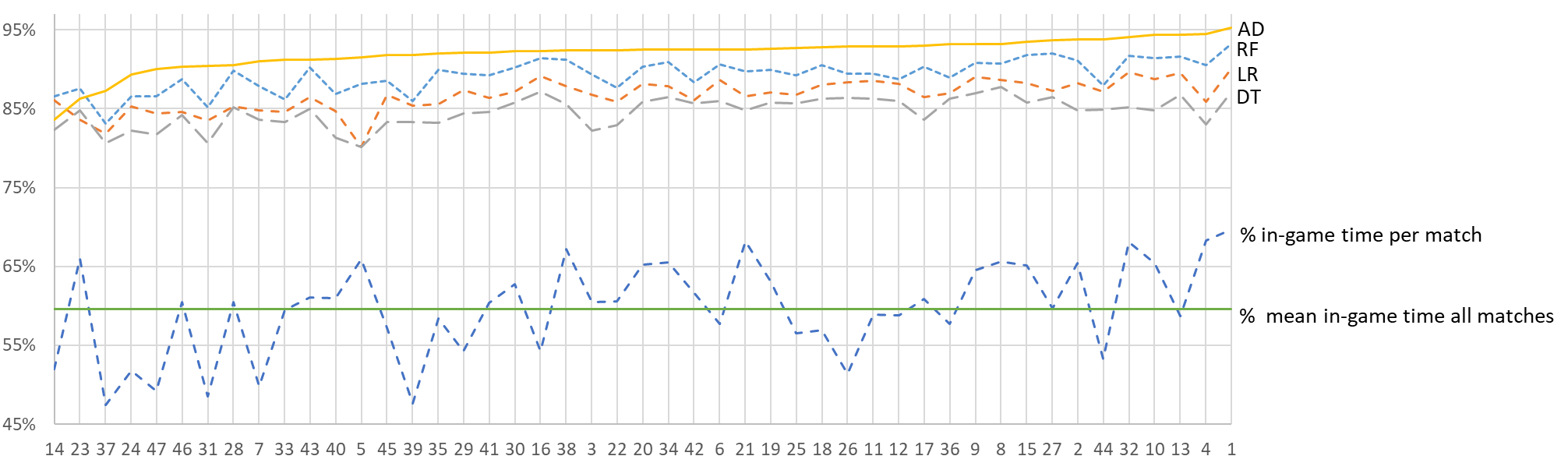


Fig. 3: Frame-by-Frame prediction accuracy in 47 test matches (x-axis) for all chosen models and the % of effective playing time per match (green) and overall mean (blue). The difference between effective playing time and the models’ prediction is the knowledge gain per match. Matches are sorted by the prediction accuracy of the AdaBoost model.


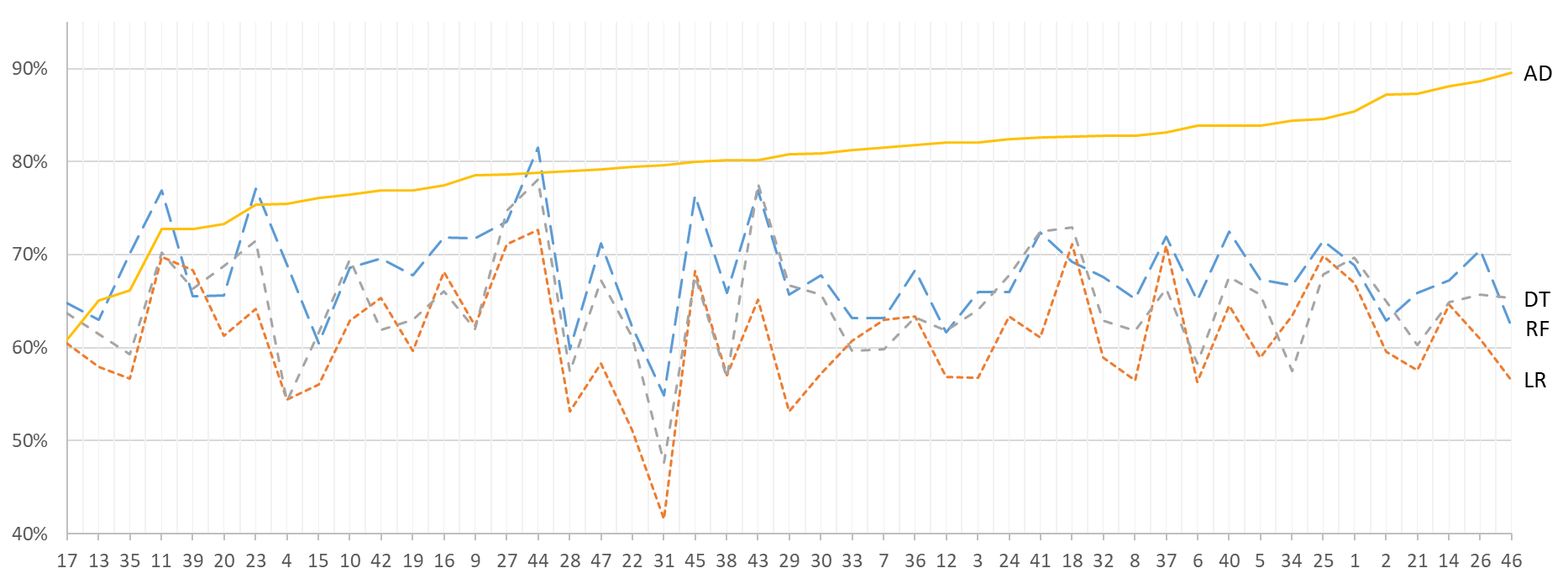


Fig. 4: F1 Scores of stoppage prediction in 47 test matches (x-axis) for all chosen models. AdaBoost (AD) model surpasses the other models in 42 of 47 matches. Matches are sorted by the F1 Score of the AD model.

| A) | 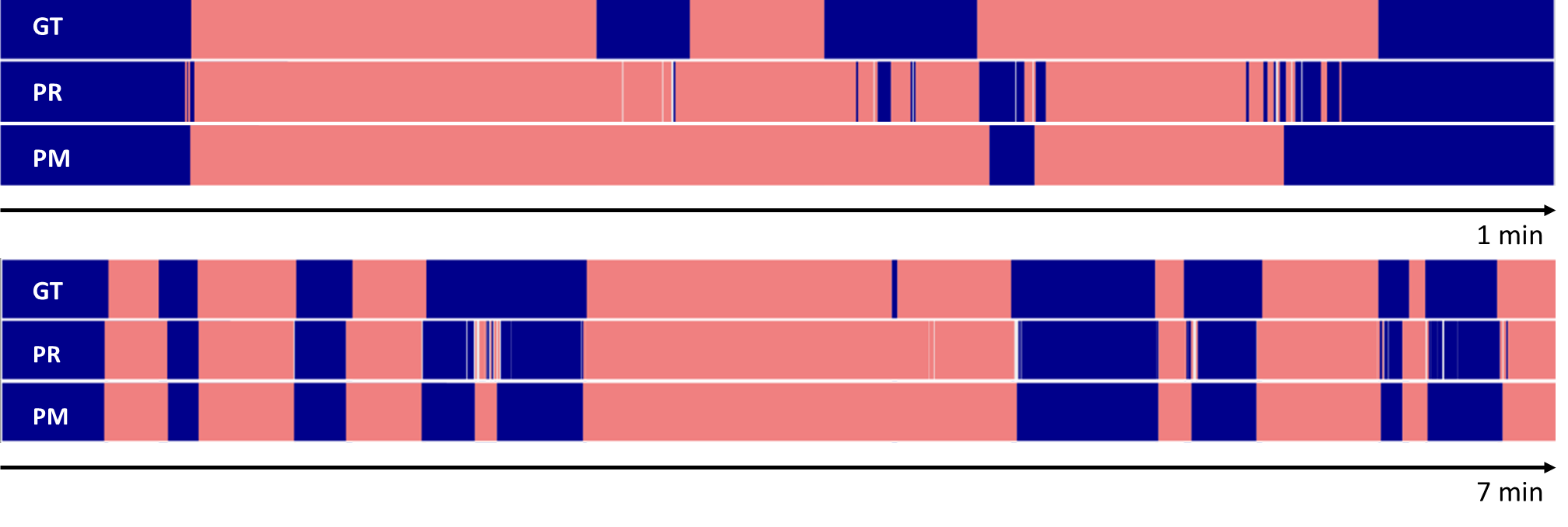 |
| --- | --- |
| B) |  |

Fig. 5: Comparison of ball status data of ground truth (GT), AdaBoost raw prediction (PR) and AdaBoost prediction after the median filter (PM) for two time sequences A) 1 minute and B) 7 minutes. The blue indicates the ball is in-play and pink means the ball is out-of-play

Tab. 4: All configured parameters for each model in the Hyperparameter Search based on scikit-learn library for Python^28^. Not here noted parameters are left to default. Explanation of the meaning of parameters can be found on scikit-learn.org. A) Randomized Search used for Random Forest and Decision Tree model. B) Grid Search used for logistic regression and AdaBoost model. The best parameters for each model are in bold.

| **A) Randomized Search** | | |
| --- | --- | --- |
|  | **Random Forest^[[1]](#footnote-1)^** | **Decision Tree^[[2]](#footnote-2)^** |
| **n_estimators** | [10, 20, 50, 100, **150**, 200] | default |
| **max_depth** | [10, 11, 12, …, **18**, 19, 20] | [10, **11**, 12, …, 18, 19, 20] |
| **min_samples_leaf** | [**1**, 2, 5, 10, 20, 50, 100, 200, 500] | [**1**, 2, 5, 10, 20, 50, 100, 200, 500] |
| **max_features** | [0.001, 0.002, 0.005, 0.01, **0.02**, 0.05, 0.1] | default |
| **min_impurity_decrease** | [0, 0.1, 0.01, 0.001, 0.0001, **0.00001**] | [0, 0.1, 0.01, 0.001, **0.0001**, 0.00001] |
|  | | |
| **B) Grid Search** | | |
|  | **Logistic regression^[[3]](#footnote-3)^** | **AdaBoost^[[4]](#footnote-4)^** |
| **penalty** | [**'l1**', 'l2'] | - |
| **C** | [0.005, 0.0051, 0.0052, …, **0.01**, …, 500] | - |
| **n_estimators** | - | [10, 20, 50, 100, 150, 200, 300, 400, 500, 600, 700, 900, 1100, 1300, 1500, 1800, **2100**, 2400, 2700, 3000] |
| **learning_rate** | - | [0.1, 0.2, **0.5**, 0.55, 0.60, 0.65, 0.70, 0.75, 1.0, 1.5, 2.0] |

1. https://scikit-learn.org/0.20/modules/generated/sklearn.ensemble.RandomForestClassifier.html [↑](#footnote-ref-1)
2. https://scikit-learn.org/0.20/modules/generated/sklearn.tree.DecisionTreeClassifier.html [↑](#footnote-ref-2)
3. https://scikit-learn.org/0.20/modules/generated/sklearn.linear_model.LogisticRegression.html [↑](#footnote-ref-3)
4. https://scikit-learn.org/0.20/modules/generated/sklearn.ensemble.AdaBoostClassifier.html [↑](#footnote-ref-4)
